# Correlation between LTR point mutations and proviral load levels among Human T cell Lymphotropic Virus type 1 (HTLV-1) asymptomatic carriers

**DOI:** 10.1186/1743-422X-8-535

**Published:** 2011-12-13

**Authors:** Walter K Neto, Antonio C Da-Costa, Ana Carolina S de Oliveira, Vanessa P Martinez, Youko Nukui, Ester C Sabino, Sabri S Sanabani

**Affiliations:** 1 Fundação Pro-Sangue, Blood Center of São Paulo, São Paulo, Brazil; 2 Department of Translational Medicine, Federal University of São Paulo, São Paulo, Brazil; 3 São Paulo inistitute of tropical medicine, São Paulo, Brazil; 4 Retrovirology Laboratory, Federal University of São Paulo, São Paulo, Brazil; 5 Department of Infectious Diseases, University of São Paulo, Sao Paulo, Brazil

## Abstract

**Background:**

In vitro studies have demonstrated that deletions and point mutations introduced into each 21 bp imperfect repeat of *Tax*-responsive element (TRE) of the genuine human T-cell leukemia virus type I (HTLV-1) viral promoter abolishes *Tax *induction. Given these data, we hypothesized that similar mutations may affect the proliferation of HTLV-1i

nfected cells and alter the proviral load (PvL). To test this hypothesis, we conducted a cross-sectional genetic analysis to compare the near-complete LTR nucleotide sequences that cover the TRE1 region in a sample of HTLV-1 asymptomatic carriers with different PvL burden.

**Methods:**

A total of 94 asymptomatic HTLV-1 carriers with both sequence from the 5' long terminal repeat (LTR) and a PvL for *Tax *DNA measured using a sensitive SYBR Green real-time PCR were studied. The 94 subjects were divided into three groups based on PvL measurement: 31 low, 29 intermediate, and 34 high. In addition, each group was compared based on sex, age, and viral genotypes. In another analysis, the median PvLs between individuals infected with mutant and wild-type viruses were compared.

**Results:**

Using a categorical analysis, a G232A substitution, located in domain A of the TRE-1 motif, was detected in 38.7% (12/31), 27.5% (8/29), and 61.8% (21/34) of subjects with low, intermediate, or high PvLs, respectively. A significant difference in the detection of this mutation was found between subjects with a high or low PvL and between those with a high or intermediate PvL (both *p *< 0.05), but not between subjects with a low or intermediate PvL (*p *> 0.05). This result was confirmed by a non-parametric analysis that showed strong evidence for higher PvLs among HTLV-1 positive individuals with the G232A mutation than those without this mutation (*p *< 0.03). No significant difference was found between the groups in relation to age, sex or viral subtypes (*p* > 0. 05).

**Conclusions:**

The data described here show that changes in domain A of the HTLV-1 TRE-1 motif resulting in the G232A mutation may increase HTLV-1 replication in a majority of infected subjects.

## Background

Human T-cell leukemia virus type I (HTLV-I) is the retrovirus responsible for adult T-cell leukemia (ATL) and the chronic neurological disorder HTLV-I-associated myelopathy/tropical spastic paraparesis (HAM/TSP) [1-4]. The virus has also been implicated in a variety of inflammatory diseases, such as uveitis [5], pulmonary alveolitis [6], Hashimoto thyroiditis [7], Sjögren's syndrome [8], and chronic arthropathy [9]. Globally, there are an estimated 10-20 million individuals are HTLV-I carriers [10]. The disease burden is unevenly distributed in endemic areas particularly in southwest Japan, the Caribbean islands, South America, and portions of Central Africa [11].

HTLV-1 is classified into seven subtypes [12]: the cosmopolitan subtype A, the Central African subtype B, the Australo-Melanesian subtype C, and subtypes D, E, F and G. The cosmopolitan subtype A is further divided into five subgroups: (A) Transcontinental, (B) Japanese, (C) West African, (D) North African, and (E) Black [13-16]. Genetic differences between HTLV-1 strains are not associated with differing clinical outcomes of HTLV-1 infections [17,18].

Similar to other retroviruses, HTLV-1 carries a diploid RNA genome comprising 9032 nucleotides that is reverse-transcribed into double-stranded DNA that integrates into host genome as a provirus. This genome contains *gag, pol *and *env *genes flanked by long terminal repeat (LTR) sequences at both 5' and 3' ends [19]. The LTR region contains enhancer/promoter genetic elements, which are critical in viral RNA transcription. A unique pX region identified between *env *and the 3' LTR encodes two key regulatory proteins Tax and Rex, as well as the various nonstructural proteins p12I, p27I, p13II, and p30II [20-22]. The Tax protein is thought to play a central role in the proliferation of infected cells and leukemogenesis because of its pleiotropic effects [23]. Tax modulates the transcription of an array of cellular genes through various cellular transcriptional signaling proteins such as NF-kB, CREB, SRF, AP-1 p53 and basic helix-loop-helix factors [11,24,25]. Tax protein has also been shown to significantly *trans*-activate HTLV RNA transcription via the viral LTR through its interaction with members of the activating transcription factor/cAMP-responsive element (CRE) binding protein bound to the viral promoter, such as ATF-1, CREB, CREB-2, or cAMP-responsive element modulator [2,26-28]. The viral promoter is located in the 5' LTR and contains three copies of a 21-bp imperfect repeat, called the Tax-responsive element 1 (TRE-1), that indirectly interacts with the Tax protein [29]. These sequences are present within the U3 region of the LTR at positions 251 to 231 (repeat I), 203 to 183 (repeat II), and 103 to 83 (repeat III) numbered-relative to the transcriptional start site. Each of the repeats is divided into three domains: A, B, and C. The central domain B of each repeat contains a conserved TGACG sequence, which shares homology with CRE, flanked by short GC-rich sequences [30,31]. Evidence from in vitro studies has demonstrated that deletions and point mutations introduced into each 21 bp repeat in the genuine viral promoter abolishes Tax induction [31,32]. We hypothesized that similar mutations may affect the proliferation of infected cells and alter the HTLV-1 PvL. Therefore, we performed a genetic analysis to compare the near complete LTR nucleotide sequences that cover the TRE regions in a sample of asymptomatic HTLV-1 carriers with different levels of PvLs.

## Materials and methods

### Patients

Peripheral blood samples (5 ml) were collected from 256 HTLV-1 positive carriers identified by the HTLV enzyme immunoassays Murex HTLV I + II (Abbott/Murex, Wiesbaden, Germany) and Vironostika HTLVI/II (bioMérieux bv, Boxtel, Netherlands) and confirmed by HTLV BLOT 2.4 (Genelabs Diagnostics, Singapore). Written informed consent was obtained from each participant. The study was approved by the local review board.

### DNA extraction and HTLV-1 proviral load determination

DNA was extracted from PBMCs using a commercial kit (Qiagen GmbH, Hilden Germany) following the manufacturer's instructions. The extracted DNA was used as a template to amplify a 158 bp fragment from the HTLV-1 *Tax *region using previously published primers [33]. The SYBR green real-time PCR assay was carried out in 25 μl PCR mixture containing 10× Tris (pH 8.3; Invitrogen, Brazil), 1.5 mM MgCl2, 0.2 μM of each primer, 0.2 mM of each dNTPs, 18.75 Units/r × n SYBR Green (Cambrex Bio Science, Rockland, ME) and 1 unit of platinum *Taq *polymerase (Invitrogen, Brazil). The amplification was performed in the Bio-Rad iCycler iQ system using an initial denaturation step at 95°C for 2 minutes, followed by 50 cycles of 95°C for 30 seconds, 57°C for 30 seconds and 72°C for 30 seconds. The human housekeeping β globin gene primers GH20 and PC04 [34] were used as an internal control. A negative, no-template control (H_2_O control) was run with every assay. Standard curves for HTLV-1 *Tax *were generated from MT-2 cells of log_10 _dilutions (from 10^5 ^to 10^0 ^copies). The threshold cycle for each clinical sample was calculated by defining the point at which the fluorescence exceeded a threshold limit. Each sample was assayed in duplicate, and the mean of the two values was considered as the copy number of the sample. The HTLV-1 proviral load was calculated as: the copy number of HTLV-1 (*Tax*) per 1,000 cells = (copy number of HTLV-1 *Tax*)/(copy number of β globin/2) × 1000 cells. The method could detect 1 copy per 10^3 ^PBMCs cells.

PvLs of *<*50, between 50 and 100, or > 100 copies/1000 PBMCs were considered to be a low, medium, or high PvL, respectively. An HTLV-1 PvL > 100 copies/1000 PBMCs have previously been shown to be associated with an increased risk of HTLV-1 disease [35].

### Amplification and sequencing of HTLV-1 LTR proviral DNA

Sequencing of the near complete LTR region of HTLV-1 proviral DNA was performed in 94 clinical isolates. PCR was performed using the primers HFL1 (39) (5' CCCAAGCTTGACAATGACCATGAGC 3') and HFL2 (782) (5'CCCGAATTCCAACTGTGTACTAAATTTC 3') and in conditions as previously described [36]. Complementary DNA strands from each amplicon were directly sequenced by cycle sequencing using the same primers used for PCR, BigDye terminator chemistry and *Taq *polymerase on an automated sequencer (ABI 3130, Applied Biosystems Inc., Foster City, CA), according to the protocols recommended by the manufacturer. The complementary sequences for each amplicon were assembled into a contiguous sequence with a minimum overlap of 30 bp with a 97-100% minimal mismatch and edited using the Sequencher program 4.7 (Gene Code Corp., Ann Arbor, MI). Sequences were then analyzed using BioEdit v.7.0.4.1 [37] and Geneious Pro v.4.8.4 (Biomatters Ltd, Auckland, New Zealand), and compared with the HTLV-1 ATK prototype (accession number J02029) [19]. A strain was considered mutant when it possessed consistent changes in its forward and reverse sequences compared to the reference wild-type strain.

### HTLV-1 genotyping

The HTLV-1 genotypes were determined by comparing the sequence of the LTR region to standard sequences from the GenBank database. HTLV subtyping was performed using the NCBI-Genotyping and REGA-Subtyping websites.

### Statistical analysis

For categorical variables, the Fisher's exact test or Chi-square test with Yate's correction was used when appropriate to analyze the association of HTLV-1 PvLs and the LTR mutational frequency. In another analysis, independent of the PvL grouping, the median PvL between subjects infected with mutant viruses to those infected with wild-type viruses was compared using the non-parametric U-Mann-Whitney test. A *p *value < 0.05 was considered significant. The data were analyzed with Stata statistical software (StataCorp, release 5.0, 1997; Stata, College Station, TX).

## Results

**Figure 1:**
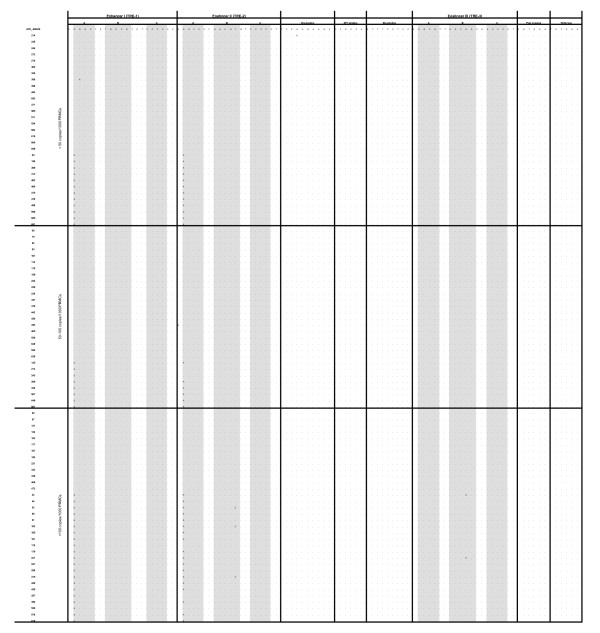
**Alignment of HTLV-1 LTR sequence elements determined from DNA amplified from 94 individuals with different concentration of proviral loads: (31 low (< 50 copies/1000 PBMCs), 29 intermediate (50-100 copies/1000 PBMCs), and 34 high (> 100 copies/1000 PBMCs)**. Domains A, B and C are indicated by a gray background, and their position in the U3 region is presented relative to ATK prototype (accession number J02029). Dots indicate nucleotide identity to the ATK prototype.

**Figure 2:**
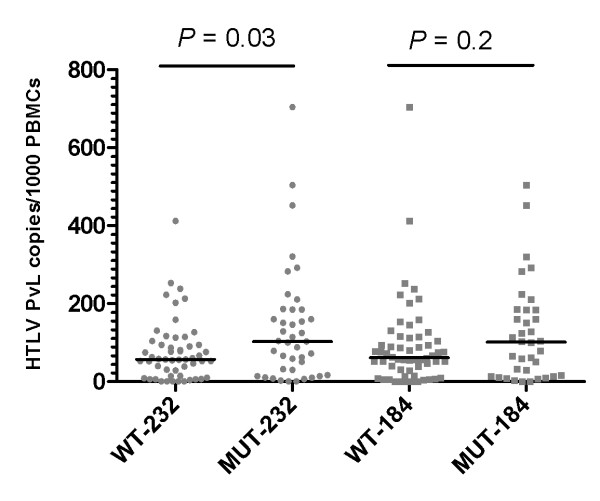
**Number of HTLV-1 subjects harboring wild-type or G232A and A184G mutant: viruses correlated with proviral loads**. Black lines represent the median proviral load for each group of subjects shown.

Out of the 256 subjects, the quantitative assay classified 71% (n = 182) in the low PvL group (median 6 copies/1000 PBMCs, range 1 to 47), 11.3% (n = 29) in the intermediate PvL group (median 63, range 51 to 98), and 17.7% (n = 45) in the high PvL group (median 160 copies/1000 PBMCs, range 103 to 708). Extracted genomic DNA from all subjects with intermediate PvL were subjected to amplification and sequencing of the near full-length HTLV-1 LTR. Due to financial constraints, 31 of the 182 genomic DNA samples from subjects with low PvL and 34 of the 45 genomic DNA samples from subjects with a high PvL were randomly selected and their HTLV-1 LTR was successfully sequenced. The demographic data from the three groups are listed in Table 1. There were no differences in the age or sex ratio between each group (*p *= 0.9 for age; *p *= 0.6 for male to female ratio). A comparison of the stratified PvLs showed no significant differences in any lymphocyte subpopulation percentages (*p *> 0.05). The transcontinental subgroup A of HTLV-1a was the major subtype; this accounted for 84% of low, 97% of intermediate, and 96% of high PvL groups. There were no differences in the subtype distribution between each group (*p *= 0.2). Mutational analyses of the non-coding regulatory sequences within the proviral LTR were conducted to determine if a correlation exist between the frequency of mutations and PvLs. These analyses revealed two relevant G232A mutation within the TRE-1 region and an A184G mutation within the TRE-2 region numbered according to the transcription start site. The G232A mutation was detected in 38.7% (12/31) of subjects with a low PvL, 27.5% (8/29) of subjects with an intermediate PvL, and 61.8% (21/34) of subjects with a high PvL (Table 1). A significant difference in detection of the G232A mutation was found between subjects with high and low PvLs and between those with high and intermediate PvLs (*p *< 0.05 both). Non statistical difference was seen between the low and intermediate PvL groups (*p *> 0.05). Thirty-six of 41 (87.8%) HTLV-1 G232A mutations were associated with a simultaneous A to G mutation at position 184 (Figure 1). The distributions of this mutation according to PvL groups was similar to the frequency of the G232A mutation being found in 38.7% (12/31) in subjects with a low PvL, 20.7% (6/29) in subjects with an intermediate PvL, and 52.9% (18/34) in subjects with a high PvL. Again, a significant difference in detection of the A184G mutation was evident between subjects with high and low PvLs and betweeen those with high and intermediate PvLs (*p *< 0.05 both), but not between the low and intermediate PvL groups (*p *> 0.05). To confirm these results, independent of the PvL grouping, a further statistical analysis using a Mann-Whitney test was performed to compare the median PvL between subjects infected with mutant viruses to those infected with wild-type viruses. This analysis revealed that while there was strong evidence for a higher PvL among HTLV-1 positive individuals with the G232A mutation than those without this mutation (42.2 *vs*. 54.4, *p *< 0.03), there was no similar association among those who were HTLV-1 positive with the A184G mutation (Figure 2). Because these two analyses did not reach the same conclusion, we considered only G232A as a potential mutation that could impact patient PvL. Other LTR motifs associated with provirus transcriptional up-regulation were inspected and were all found similar to a wild-type genotype with few scattered natural point mutations (Figure 1).

**Table 1:** Clinical and virological characteristics by proviral load levels in subjects with available LTR sequences

Characteristics	HTLV-1 proviral copies/1000 PBMCs		
	
	< 50 (n 31)	50-100 (n 29)	> 100 (n 34)
Median age*. (range)	50 (26-71)	51 (24-70)	59.5 (21-95)

Sex			

Male (%)	12 (39)	9 (31)	15 (44)

Female (%)	19 (61)	20 (69)	19 (56)

Lymphocyte subpopulations			

% CD4 cells	42.9	45.2	46.8

% CD8 cells	28.2	27.3	29.1

% CD25 cells	20.3	22.6	29.8

Subtype A (%)			

Subgroup A (%)	26 (84)	28 (97)	32 (96)

Subgroup B (%)	5 (16)	1(3)	2 (4)

Mutation			

TRE-1 G232A (%)	12 (38.7)	8 (27.5)	21 (61.8)

TRE-1 A184G (%)	12 (38.7)	6 (20.7)	18 (52.9)

*Years

## Discussion

In the present cross-sectional, retrospective study, we investigated mutations in the LTR region of HTLV-1 and compared their association with asymptomatic carriers PvL. Our results showed that mutations were relatively rare. However, a solitary, natural mutation at position 232 was significantly associated with HTLV-1 infected subjects' PvL. The G232A mutation is located in domain A of the TRE-1 motif that contains non-consensus CREB response elements and is involved in *Tax*-activated and basal LTR expression. Although the functional importance is unclear, it is possible that the G232A mutation in the TRE1 element may enhance indirect binding to *Tax *via the CREB family of cellular transcription factors, thereby promoting the proliferation of infected cells and resulting in an increase in patient PvLs. However, the validity of this hypothesis needs to be investigated in an independent series of experiments comparing the expression and activation of the *Tax *gene in HTLV-1-infected T cells with or without the G232A mutation. Previous reports have shown that mutations that abolish *Tax *effects are all localized in the CREB of the 21 bp repeats [38]. Montagne *et al*. demonstrated that mutations introduced into domain A or C can severely impair the ability of the Tax protein to transactivate different promoters [31]. Taken together, these data suggest that the TRE-1 element plays an important role in the activation of HTLV-1 gene expression.

In our study, the G232A mutation was associated with HTLV-1 PvL. Although the G232A mutation was detected at a higher frequency in asymptomatic HTLV-1 carriers in this study, the frequency of such a mutation is expected to be higher in patients with overt clinical disease, because the PvLs of HTLV-1-infected patients correlates with clinical outcome [35]. It is important to note that our study is limited by the nature of its cross-sectional design, and these results imply only a correlation; therefore, no inferences can be made as to the evolution of the wild-type viruses during chronic infection. Therefore, longitudinal study designs are required to address these issues and also to determine if the G232A mutations was already present in patients with advanced disease before they were diagnosed a chronic infection.

Although the G232A mutation was detected in some subjects with a low PvL in this study, further study is needed to determine if this mutation is predictive of an increase in PvL in patients who possess the mutation but do not have an elevated PvL yet.

As shown in Figure 1, the G232A and A184G mutations occurred together in almost all instances, most likely because an HTLV strain with both mutations has a higher selective advantage. Interestingly, none of the subjects examined contain only the A184G mutations. Therefore, additional experimental supports are needed to rule out the potential importance of the A184G mutations and functional connection between A184G and G232A mutations.

Our analysis was limited to HTLV-1 subgroup A because other subtypes are rare in our patients. Whether similar results will be obtained for other subgroups, is of interest.

In conclusion, the results described here suggest the G232A mutation in domain A of the TRE-1 motif may increase HTLV-1 replication in the majority of infected subjects.

## List of abbreviations

HTLV-1: Human T Cell Lymphotropic Virus Type 1; TRE: Tax-responsive element; PvL: Proviral load; LTR: Long terminal repeat; ATL: Adult T-cell leukemia; HAM/TSP: HTLV-I-associated myelopathy/tropical spastic paraparesis; CREB: Transcriptional activity of cAMP binding protein; NFkB: Nuclear factor kappa B; SRF: Serum Response Factor; AP-1: Activator protein; CRE: CAMP-responsive element; ATF-1: Activating transcription factor 1.

## Competing interests

The authors declare that they have no competing interests.

## Authors' contributions

WKN and ACC conceived and designed the study, did the data analysis of the sequences. ACSO and VPM conducted the characterization of the LTR sequences and data analysis of the sequences. YN was responsible of the clinical management of patients, acquisition of data; ECS was responsible of the scientific revision, discussion and interpretation of data. SSS wrote the manuscript and directed the study. All authors read and approved the final manuscript.
